# SmCL3, a Gastrodermal Cysteine Protease of the Human Blood Fluke *Schistosoma mansoni*

**DOI:** 10.1371/journal.pntd.0000449

**Published:** 2009-06-02

**Authors:** Jan Dvořák, Susan T. Mashiyama, Mohammed Sajid, Simon Braschi, Melaine Delcroix, Eric L. Schneider, Wilson H. McKerrow, Mahmoud Bahgat, Elizabeth Hansell, Patricia C. Babbitt, Charles S. Craik, James H. McKerrow, Conor R. Caffrey

**Affiliations:** 1 Sandler Center for Basic Research in Parasitic Diseases, California Institute for Quantitative Biosciences, University of California San Francisco, San Francisco, California, United States of America; 2 Departments of Biopharmaceutical Sciences and Pharmaceutical Chemistry, California Institute for Quantitative Biosciences, University of California San Francisco, San Francisco, California, United States of America; 3 Leiden University Medical Centre, afd. Parasitologie, Leiden, The Netherlands; 4 Therapeutical Chemistry Department, Infectious Diseases and Immunology Laboratory, The Road to Nobel Project, The National Research Center, Dokki, Cairo, Egypt; McGill University, Canada

## Abstract

**Background:**

Blood flukes of the genus *Schistosoma* are platyhelminth parasites that infect 200 million people worldwide. Digestion of nutrients from the host bloodstream is essential for parasite development and reproduction. A network of proteolytic enzymes (proteases) facilitates hydrolysis of host hemoglobin and serum proteins.

**Methodology/Principal Findings:**

We identified a new cathepsin L termed SmCL3 using PCR strategies based on *S. mansoni* EST sequence data. An ortholog is present in *Schistosoma japonicum*. SmCL3 was heterologously expressed as an active enzyme in the yeast, *Pichia pastoris*. Recombinant SmCL3 has a broad pH activity range against peptidyl substrates and is inhibited by Clan CA protease inhibitors. Consistent with a function in degrading host proteins, SmCL3 hydrolyzes serum albumin and hemoglobin, is localized to the adult gastrodermis, and is expressed mainly in those life stages infecting the mammalian host. The predominant form of SmCL3 in the parasite exists as a zymogen, which is unusual for proteases. This zymogen includes an unusually long prodomain with alpha helical secondary structure motifs. The striking specificity of SmCL3 for amino acids with large aromatic side chains (Trp and Tyr) at the P2 substrate position, as determined with positional scanning-synthetic combinatorial library, is consistent with a molecular model that shows a large and deep S2 pocket. A sequence similarity network (SSN) view clusters SmCL3 and other cathepsins L in accordance with previous large-scale phylogenetic analyses that identify six super kingdoms.

**Conclusions/Significance:**

SmCL3 is a gut-associated cathepsin L that may contribute to the network of proteases involved in degrading host blood proteins as nutrients. Furthermore, this enzyme exhibits some unusual sequence and biophysical features that may result in additional functions. The visualization of network inter-relationships among cathepsins L suggests that these enzymes are suitable ‘marker sequences’ for inclusion in future phylogenetic analyses.

## Author Summary

Parasitic infection caused by blood flukes of the genus *Schistosoma* is a major global health problem. More than 200 million people are infected. Identifying and characterizing the constituent enzymes of the parasite's biochemical pathways should reveal opportunities for developing new therapies (i.e., vaccines, drugs). Schistosomes feed on host blood, and a number of proteolytic enzymes (proteases) contribute to this process. We have identified and characterized a new protease, SmCL3 (for *Schistosoma mansoni* cathepsin L3), that is found within the gut tissue of the parasite. We have employed various biochemical and molecular biological methods and sequence similarity analyses to characterize SmCL3 and obtain insights into its possible functions in the parasite, as well as its evolutionary position among cathepsin L proteases in general. SmCL3 hydrolyzes major host blood proteins (serum albumin and hemoglobin) and is expressed in parasite life stages infecting the mammalian host. Enzyme substrate specificity detected by positional scanning-synthetic combinatorial library was confirmed by molecular modeling. A sequence analysis placed SmCL3 to the cluster of other cathepsins L in accordance with previous phylogenetic analyses.

## Introduction

Proteases (proteolytic enzymes, peptidases) provide essential functions in all life forms [1]. Proteases function as key elements of parasitism including hatching, excystment, tissue/cell invasion, nutrient acquisition and immune evasion [2],[3]. For trematode parasites causing diseases of medical and veterinary importance, proteases operate at the host-parasite interface facilitating migration, digestion of host proteins and probably immune evasion [3],[4].

Within the family Schistosomatidae, three major species infect more than 200 million people worldwide [5]. After penetration of human skin by aquatic larvae (cercariae), immature parasites (schistosomula) migrate within the vascular system to the final predilection site where females produce eggs upon maturation. Parasite development and fecundity rely on nutrients ingested from the host bloodstream. A network of proteases with differing catalytic mechanisms “Clans” as described in the MEROPS database (http://merops.sanger.ac.uk/) has been identified in the schistosome gut and facilitates digestion of proteins to absorbable peptides and amino acids [6]–[8]. For *Schistosoma mansoni*, the component proteases thus far characterized include Clan CA *S. mansoni* cathepsin B1 (SmCB1), SmCL1(SmCF) and SmCL2, SmCC, a Clan CD asparaginyl endopeptidase (SmAE), a Clan AA aspartic protease SmCD and a Clan MF leucine metallo-aminopeptidase [7],[9]. Proteolytic networks associated with host protein degradation and comprising the same protease clans have been described for other parasitic platyhelminths [4] and are conserved across phylogenetically diverse organisms such as *Plasmodium* [10], nematodes [11] and arthropods [12].

Given their central importance in the biology of the parasite, gut proteases have been tested as vaccine candidates for disease prophylaxis [13],[14] and are potential chemotherapeutic targets [15],[16]. As immunodominant antigens, some schistosome gut proteases have been experimentally proven as serodiagnostic antigens [17].

In this study, we have identified and characterized a new cathepsin L in *S. mansoni*, SmCL3. From the original expressed sequence tag (EST) [18] we have cloned and sequenced the full-length open reading frame (ORF), and heterogeneously expressed the enzyme in the yeast, *Pichia pastoris*. The hydrolytic activity and specificity of the recombinant protease were characterized using active site-directed affinity probes, peptidyl substrates and a positional scanning-synthetic combinatorial library (PS-SCL). Monospecific antibodies localized SmCL3 to the gut. Distinct from SmCL1 and SmCL2, the N-terminus of the SmCL3 zymogen is extended by approximately 30 amino acids, and the enzyme exists primarily as a zymogen in the parasite rather than as a fully processed mature enzyme. Sequence similarity clustering and visualization using Cytoscape [19] places SmCL3 in the metazoan cathepsin L cluster along with SmCL2 and cathepsins L from the liver fluke, *Fasciola* spp.. This cluster is distinct from a second group of cathepsins F that includes SmCL1 and those from other trematode parasites such as *Opisthorchis*, *Paragonimus* and *Clonorchis*.

## Materials and Methods

### Schistosome material

*S. mansoni* (a Puerto Rican isolate) is maintained in the laboratory by cycling between the freshwater snail, *Biomphalaria glabrata*, and the golden hamster, *Mesocricetus auratus*. Hamsters are maintained in barrier facilities as approved by the Institutional Animal Care and Use Committee of the University of California San Francisco (IACUC). All animal experiments were carried out in accordance with the same protocols approved by the IACUC. Infections with *S. mansoni* are initiated by subcutaneous injections of 500–1000 cercariae. At 6–7 weeks post-infection, hamsters are euthanized with intra-peritoneal injections of sodium pentobarbital (50 mg/kg), and adult worms harvested by reverse perfusion of the hepatic portal system [20] in RPMI 1640 medium (Invitrogen). Complete Medium 169 containing 5% fetal calf serum and 1% ABAM (Antibiotics/Antimycotics: Sigma-Aldrich), was used to maintain immature (schistosomula) and adult worms *in vitro* [21]. For preparation of schistosomula, cercariae were harvested from the infected snails by light induction for 1 h, and chilled on ice in a 50 ml falcon tube. The water was poured off and replaced with chilled incomplete Medium 169 (without serum) in preparation for shearing of tails, a method modified from Colley and Wikel [22]. Cercariae were then passed back and forth 15 times between two 10 ml syringes connected by a double-headed 22 gauge needle. Upon deposition into a 5 cm Petri dish, the lighter tails were separated from heads by swirling and aspiration with a Pasteur pipet. The nascent schistosomula were then collected and washed three times in Incomplete Medium 169. After recovery from hamsters, adult worms were washed 5 times in incomplete Medium 169. Both schistosomula and adults were maintained in complete Medium 169 under a 5% CO_2_ atmosphere at 37°C. Miracidia (the stage infective to the snail) were prepared from eggs trypsinized from infected liver tissue and hatched in freshwater.

### Sequencing and cloning

A partial sequence encoding the cathepsin L3 was obtained from the *S. mansoni* EST database [18]. Gene-specific primers were used to verify the cathepsin L3 gene sequence. Briefly, *S. mansoni* mRNA was isolated from adult worms using the FastTrack 2.0 isolation kit (Invitrogen), and single strand cDNA was prepared using Superscript III Reverse Transcriptase (Invitrogen) with an oligo-dT_18_ primer. Purified cDNA was then used as template for PCR using Taq Platinum polymerase (Invitrogen) and gene-specific primers, SmCL3frd1 (5′-GCCTGGCTCTGTAAATGTTGAG -3′) and SmCL3rev1 (5′- CATATGGATAGGAAATCTCAGAATC -3′). A 350 bp product was amplified and subsequently cloned into pCR 2.1-TOPO cloning vector (Invitrogen) for propagation in *E. coli*. Five positive clones were analyzed for sequence verification.

Full-length cathepsin L gene was retrieved by rapid amplification of cDNA ends (RACE) using the GeneRacer Kit (Invitrogen) according to the manufacturer's instructions. Gene specific primers for 3′ RACE were SmCL3 3′RACE frd1 (5′- GTTGCGTGGATATAAAGTCACTAG -3′) and SmCL3 3′RACE frd2 (5′- GCTATCAGACATAAAGGGTCGAC -3′). For 5′RACE the primers were SmCL3 5′RACE rev1 (5′- GTCGACCCTTTATGTCTGATAGC -3′) and SmCL3 5′RACE rev2 (5′- CTAGTGACTTTATATCCACGCAAC -3′). Final amplicons were cloned into pCR 2.1-TOPO cloning vector and sequenced.

To verify the entire ORF sequence, PCR incorporated Platinum Taq polymerase, cDNA from adult worms and primers directed to the 5′ and 3′ ends of the SmCL3 gene. The resulting amplicons were cloned into pCR 2.1-TOPO cloning vector and 10 randomly selected positive *E. coli* clones were sequenced.

### Stage-specific expression profiling of SmCL3 using quantitative PCR

Total RNA was extracted from *S. mansoni* eggs, daughter sporocysts extracted from hepatopancreases of snails patent for infection, cercariae, newly transformed schistosomula (incubated *in vitro* for 24 h), and adult worms using Trizol reagent according to the manufacturer's instructions (Invitrogen). The precipitation step was omitted and RNA from the aqueous phase was purified using the RNA Isolation Kit (Stratagene) according to the manufacturer's instructions. The concentration of RNA was determined by absorbance at 260 nm using a ND-1000 Spectrophotometer (NanoDrop). Single-stranded cDNA was synthesized from 1 µg of total RNA using SuperScript III reverse transcriptase (Invitrogen) and an oligo d(T)_18_ reverse primer according to the manufacturer's protocol, and the resulting cDNA was purified. Quantitative PCR (qPCR) was carried out using the SYBR-green MasterMix Plus Kit (Eurogentech) with 1 µl of purified cDNA and each of 2 sets of forward and reverse primers (0.1 µl; 2.4 µM each; Table S1) that had been designed using the Primer 3 software (http://frodo.wi.mit.edu/cgi-bin/primer3/primer3_www.cgi, [23]) and designed to amplify 150–250 bp fragments.

Triplicate reactions were carried out in a final volume of 25 µl in 96 well plates in a MX 3005P Real-Time PCR cycler (Stratagene). The amplification profile consisted of an initial hot start (95°C for 10 min) followed by 40 cycles comprising 95°C for 30 s, 55°C for 1 min and 72°C for 30 s. The ROX dye and *S. mansoni* cytochrome C oxidase I (SmCyCOx) (GenBank AF216698, [24]) were always used as a reference dye and reference gene, respectively. Upon completion of the amplification, the dissociation curve was examined for potential primer dimerization. The cycle threshold (CT) values were averaged and the standard deviation was determined. The relative expression levels were calculated using the formula 2 ^−(SmCyCOx CT – Gene of interest CT)^ [25].

### Production of recombinant SmCL3 in *Pichia pastoris*

The primary amino acid sequence coding the SmCL3 gene was analyzed by SignalP (http://www.cbs.dtu.dk/services/SignalP/; [26]) to identify the predicted starting position of the proenzyme which was then amplified with Pfx DNA polymerase (Invitrogen) using the cloning primers SmCL3picZB frd, 5′-GATACTGCAGATTCTGGTTTCAGAAAGTGGTC-3′ (*Pst I* restriction site underlined; *note*: the Kex 2 yeast protease processing site is placed upstream in the expression pPICZ αB vector) and SmCL3picZB rev, 5′-TAAGCGGCCGC *TCA*TACTAGAGGGTATGAAGCCGCACTGGCA-3′ (*Not I* restriction site underlined, termination codon in italic). Alternatively, a histidine-tagged reverse cloning primer, SmCL3picZB revHis, 5′-TAAGCGGCCGC *TCA* **CATCATCATCATCATCAT**TACTAGAGGGTATGAAGCCGCACTGGCA-3′ (*Not I* restriction site underlined, termination codon in italic, 6×His-tag in bold), was used to amplify a C-terminal histidine- tagged form (SmCL3-his) to facilitate subsequent purification and concentration of the SmCL3 expression product. The resulting PCR products were sub-cloned into the expression vector pPICZ αB (Invitrogen), as previously described [27] and sequences verified. Transformation of *P. pastoris* and protein expression were carried out as described previously [27],[28].

### Purification of recombinant SmCL3

The induction yeast medium containing recombinant enzyme was filtered (0.45 µm), lyophilized and stored at −20°C until use. The powder was resuspended to 10% of the induction volume, and desalted using PD-10 columns (GE-Healthcare) and eluted in 50 mM sodium phosphate (pH 6.0) for non his-tagged enzyme, or 50 mM sodium phosphate, pH 7.5, 500 mM NaCl for SmCL3-his SmCL3-his was purified further on a HisTrap 5 ml column (GE-Healthcare). The column was equilibrated with 50 mM sodium phosphate, 500 mM NaCl, pH 8.0. Enzyme sample was loaded and the column washed with 25 ml of 50 mM sodium phosphate, 500 mM NaCl, 20 mM imidazole, pH 6.0 and eluted in same buffer containing 500 mM imidazole. Salt and imidazole were then removed by buffer exchange on an Amicon Ultra 10 kDa spinning column by 5 wash and centrifugation steps into 50 mM citrate, 100 mM sodium phosphate buffer, pH 6.0.

The presence of active recombinant enzyme was verified by protease activity assay (see Protease activity assays) and by SDS-PAGE gels with protein visualized either with iodinated clan CA affinity label ^125^I-DCG-04 (see Active site labeling) or with SafeStain protein dye (Invitrogen). The cleavage sites used to generate the active recombinant enzyme were identified by N-terminal protein sequencing (Protein and Nucleic Acid Facility, Stanford University). The recombinant enzyme was stored at −20°C.

To determine glycosylation status, recombinant SmCL3 activity was inhibited for 30 min at RT with 10 µM K11777 and deglycosylated using endoglycosidase H (Endo-H, Roche) according to the manufacturer's protocol. Samples were then resolved by 15% SDS-PAGE.

### Active site labeling

The specific irreversible affinity probe for Clan CA cysteine proteases, ^125^I-DCG-04 [29] was used to label the active site of recombinant SmCL3 at pH 6.0, as previously described [30]. Prior to radiolabeling, control samples were incubated for 20 min in the presence of 10 µM of the Clan CA cysteine protease inhibitor E-64 (L-trans-epoxysuccinyl-leucylamide-(4-guanido)-butane; Sigma) or preheated at 70°C. Labeled SmCL3 samples were resolved by SDS-PAGE (15% Tris-HCl Criterion gel; Biorad) and visualized by autoradiography using a Typhoon Trio 8600 Variable Mode Imager (GE Healthcare).

### Protease activity assays, kinetics and inhibition constants

Proteolytic activity was measured with the synthetic fluorogenic dipeptidyl substrate Z-Phe-Arg-AMC (benzyloxycarbonyl-phenylalanylarginine-7-amido-4-methylcoumarin; Bachem). Assays were performed in black 96-well plates as described previously [28]. Briefly, recombinant SmCL3 enzyme was pre-incubated for 10 min at RT (room temperature) in 50 mM citrate, 100 mM sodium phosphate, pH 3.0–8.0 or 100 mM glycine, pH 7.0–11.0. All buffers contained 100 mM NaCl and 2 mM dithiothreitol (DTT) in a final volume of 100 µl. The reactions were started by adding 100 µl of the same buffer solution containing 40 µM Z-Phe-Arg-AMC. Release of free AMC was measured at excitation and emission wavelengths of 355 and 460 nm, respectively, in a Labsystems Fluoroskan II fluorescent plate reader (Thermo Electron Corporation).

For pH stability assays, recombinant SmCL3 samples were incubated in 50 mM citrate, 100 mM sodium phosphate, 2 mM DTT, pH 3.0–8.0 at 37°C for 1 h. Enzyme activities were analyzed at pH 6.0 using fluorescent dipeptidyl substrate Z-Phe-Arg-AMC and active site labeling with ^125^I-DCG-04.

The *K*_m_ value and *k*_cat_ (turnover rate) for SmCL3 with Z-Phe-Arg-AMC were determined by nonlinear regression analysis Prism 4 (GraphPad). Rates were obtained from substrate concentrations (0.2–150 µM) with a fixed enzyme concentration of 3 nM. Assays were performed in black 96-well plates in 50 mM citrate, 100 mM sodium phosphate, pH 6.0 at a final volume of 200 µl. Release of free AMC was measured at 25°C in a Flex Station fluorescent plate reader (Molecular Devices).

Kinetic analyses with irreversible cysteine protease inhibitors were performed as previously described [31]. Enzyme (∼3 nM) in 100 µL 50 mM citrate, 100 mM sodium phosphate, pH 6.0 (see above), was added to inhibitor dilutions in 100 µL of the same assay buffer containing 25 µM Z-Phe-Arg-AMC. Progress curves were recorded for 5 min in the Flex Station fluorescent plate reader at 25°C (less than 5% of substrate consumed) over a range of dilutions (0.5, 0.4, 0.3, 0.2, 0.1, 0.05, and 0 µM) of inhibitors the cysteine protease inhibitors E-64 or K11777 (*N*-methyl piperazine-ureaphenylalanyl-homophenylalanyl-vinylsulfone-benzene [32],[33] dissolved in DMSO (final DMSO in assay was 0.5%). Inhibitor dilutions giving simple exponential progress curves over a wide range of *k*_obs_ (first order observed inhibition constant) with r∧2 values ≥ to 0.9 were used to determine kinetic parameters. The value of *k*_obs_, the rate constant for loss of enzyme activity, was determined from an equation for pseudo first order dynamics using Prism4 software (GraphPad). When *k*_obs_ varied linearly with inhibitor concentration, *k*_ass_ (complex formation constant) was determined by linear regression analysis [34]. If the variation was hyperbolic, indicating saturation inhibition kinetics, *k*_inact_ (maximal inactivation rate constant) and *K*_i_ (inhibition constant) were determined from an equation describing a two step irreversible inhibitor mechanism (*k*_obs_ = *k*_inact_ [I]o/([I]o+*K*_i*_ (1+[S]o/*K*_m_))) and nonlinear regression analysis using Prism. 4.

### Incubation of recombinant SmCL3 with protein substrates

Recombinant SmCL3 (∼100 nM) was incubated overnight at 37°C with bovine albumin or bovine hemoglobin (1 mg/ml; Sigma) in 50 mM citrate, 100 mM sodium phosphate, 2 mM DTT, pH 3.0–10.0. After incubation, a 20 µl sample was resolved by 10% Bis-Tris NuPage Novex gel with MES buffer running buffer (Invitrogen).

### Subsite specificity profiling by positional scanning-synthetic combinatorial library (PS-SCL)

PS-SCL were employed as previously described [35]. All 20 amino acids were incorporated in tetrapeptides where cysteine was omitted and norleucine included. Assays involving either SmCL3 or SmCL3-his were carried out in black 96-well microtiter plates at pH 6.0, as described previously [35],[36]. Release of 7-amino-4-carbamoylmethylcoumarin (ACC) was measured in a Perkin-Elmer LS50B luminescence spectrometer with excitation and emission wavelengths set to 380 and 460 nm, respectively.

### Production of mouse polyclonal antibodies to recombinant SmCL3

One mg of purified recombinant SmCL3-his was resolved by SDS-PAGE (12% Tris-HCl Criterion gel; Biorad). Gels were briefly stained in SimplyBlue Safe Stain to visualize the SmCL3 protein band and then washed with water. The protein band was excised and homogenized in sterile saline using a glass homogenizer. Five Swiss-Webster mice were injected with a 100 µl mixture of antigen and adjuvant 4 times at 14 day intervals. The first injection was administered intraperitoneally in Freunds Complete Adjuvant (Sigma) in a ratio 3∶1. Three subsequent subcutaneous injections contained antigen in TiterMax Gold adjuvant (Sigma) at a 2∶1 ratio. For control sera, blood samples were withdrawn from mice receiving acrylamide samples alone. Seven days after the last injection, mice were euthanized and exsanguinated. After clotting, serum was separated from blood cells and then the IgG fraction isolated using a HiTrap Protein G column (GE-Healthcare), according to the manufacturer's protocol.

### Immunoblotting

For immunoblotting, *S. mansoni* soluble protein extracts were prepared by sonication in 50 mM citrate, 100 mM phosphate, pH 5.0 over an ice bath in the presence of Protease Inhibitor Cocktail (Sigma). After brief centrifugation at 8 000 *g* for 5 min at 4°C, supernatants containing soluble proteins were collected. Extracts (20 µg per well) and recombinant SmCL3 were resolved by SDS-PAGE (15% Tris-HCl Criterion gels) and transferred onto a PVDF membrane (Biorad). Membranes were blocked overnight at 4°C in 5% non-fat dry milk in Tris-buffered saline containing 0.1% Tween 20 (TBS-T) and washed 3×5 min in TBS-T. After washing, membranes were incubated for 1 h with anti-SmCL3 or control purified polyclonal IgG (1∶1000) in TBS-T. Membranes were then washed 3×15 min in TBS-T and incubated for 1 h with anti-mouse IgG-HRP conjugate (GE Healthcare) at a dilution of 1∶2000. After washing in TBS-T 3×15 min, followed by a single wash in TBS for 5 min, membranes were developed using an enhanced chemiluminescent kit (ECL Western Blotting Detection Reagents, GE Healthcare) according to the manufacturer's instructions. Immunoreactivity was visualized by exposure to the SuperRX Medical X-Ray Film (Fuji).

### Immunolocalization

Perfused adult *S. mansoni* worms were fixed in 0.1% glutaraldehyde in PBS, pH 7.4 at RT for 2 h, washed 3×15 in PBS, pH 7.4 and stored at 4°C prior to use. Samples were then embedded in JB-4 (Polyscience), sectioned at 2.5 µm, placed on glass slides and dried at 60°C for 5 min. Incubation with mouse control or anti-SmCL1 IgG antibodies at 1∶200 dilutions in TBS-T and secondary Alexa Fluor 594 anti-mouse IgG (Invitrogen) was carried out as described [37]. Localization was observed using a laser scanning microscope LSM 510 META (Carl Zeiss).

### Size exclusion chromatography of *S. mansoni* soluble protein extract

Soluble extract from adult worms was prepared as described above. The extract was size fractioned using pre-equilibrated column Superdex 200 (GE- Healthcare) according to manufacture's instructions. Eluted fractions were resolved by SDS-PAGE (15% Tris-HCl Criterion gel, Biorad) and transferred onto a PVDF membrane (Biorad) and SmCL3 was detected by Western blot analysis.

### 3D structural modeling of SmCL3

The SmCL3 protein sequence was used as a query in a web-based blastp at http://blast.ncbi.nlm.nih.gov [38] search of the Protein Data Bank (PDB; http://www.rcsb.org/pdb) using the default setting of filtering low-complexity regions. The fourth best hit was used as the template for modeling because this hit had a good E-value and also included an inhibitor complexed with the protein, which improves modeling results. The template was human cathepsin V complexed with vinyl sulfone inhibitor K11777 [32],[33], pdb ID 1FH0, (with identical chains A and B, solved to 1.6 Å resolution). The BLAST alignment of SmCL3 and 1FH0 had 59% sequence identity (135/227 residues), E-value = 2e-74. The alignment from the BLAST search was used with the homology modeling program PLOP [39]. In order to show the active site as a substrate would likely bind, views of the model were generated in Chimera [40] as follows: The template 1FH0, chain A, was aligned to the SmCL3 model in Chimera using the Matchmaker tool. The template was then hidden except for the inhibitor. The catalytic Cys^172^ and His^317^ were colored yellow and blue, respectively. The residues in the S2 binding pocket that are ≤5 Å from the inhibitor are shown in ball-and-stick format. The predicted residues in this pocket are identical to those in the 1FH0 template (cathepsin V) except for one residue which is Leu^216^ in SmCL3 (colored light green) and Phe^69^ in 1FH0. Other important active site residues Gln^166^ and Asn^337^ aligned closely with the same corresponding residues in 1FH0 (not highlighted in the model).

To analyze SmCL3 prodomain structure, protein sequence was also imported into the protein modeling program interface Maestro (Maestro 8.5207, Schrodinger, LLC), and the secondary structure prediction program PSIpred [41] run on the sequence through the Maestro interface using the Prime application (Prime 2.0208, Schrodinger, LLC). Secondary structure prediction programs such as PSIpred are about 75% accurate (http://cubic.bioc.columbia.edu/eva/sec/res_sec.html).

### Sequence similarity network (SSN) depicting relationships among SmCL3 and other cathepsin L-like genes

SmCL3 was queried against the UniRef100 database (http://www.ebi.ac.uk/uniref/) [42] of non-redundant protein sequences using the program blastp [38]. A perl script was then used to select 1025 sequence hits scoring at E-value≤1e^−30^ and where the alignment length was at least 80% of the query length. The sequences were filtered to a set of 297 sequences ≤60% identical to each other using the program CD-HIT [43]. An all vs. all blastp search of these representative sequences was then performed to find sequence similarity relationships between all 297 proteins. Perl scripts were used to parse the species names from the UniRef IDs and to key species to class using data from NCBI Taxonomy (http://www.ncbi.nlm.nih.gov/Taxonomy). The resulting data of sequence similarity relationships and node labels were formatted, colored by class and visualized using sequence similarity networks (SSNs) for visualization of relationships across diverse protein superfamilies [44] in the ‘organic’ layout with Cytoscape v2.4.1 [19]. An E-value cutoff threshold of 1e^−60^ was used for drawing edges between sequences. Cytoscape is an open source bioinformatics software platform for visualizing many types of biological networks (http://www.cytoscape.org/index.php). In the ‘organic’ view, each representative sequence is displayed as a colored “node” with lines connecting nodes signifying a BLAST E-value relationship better than the cutoff value. The 247 nodes that formed clusters are shown; more highly interconnected nodes have shorter edges than less well-connected nodes. To aid interpretation of the output, the nodes were also colored to correspond to a super kingdom classification proposed by Simpson and Roger [45]. For details about included gene sequences see supporting Cytoscape data (Fig. S1; Note: you have to download Cytoscape v2.4.1 program at http://www.cytoscape.org/index.php).

## Results

### SmCL3—a cathepsin L with unusual sequence features

**Figure 1:**
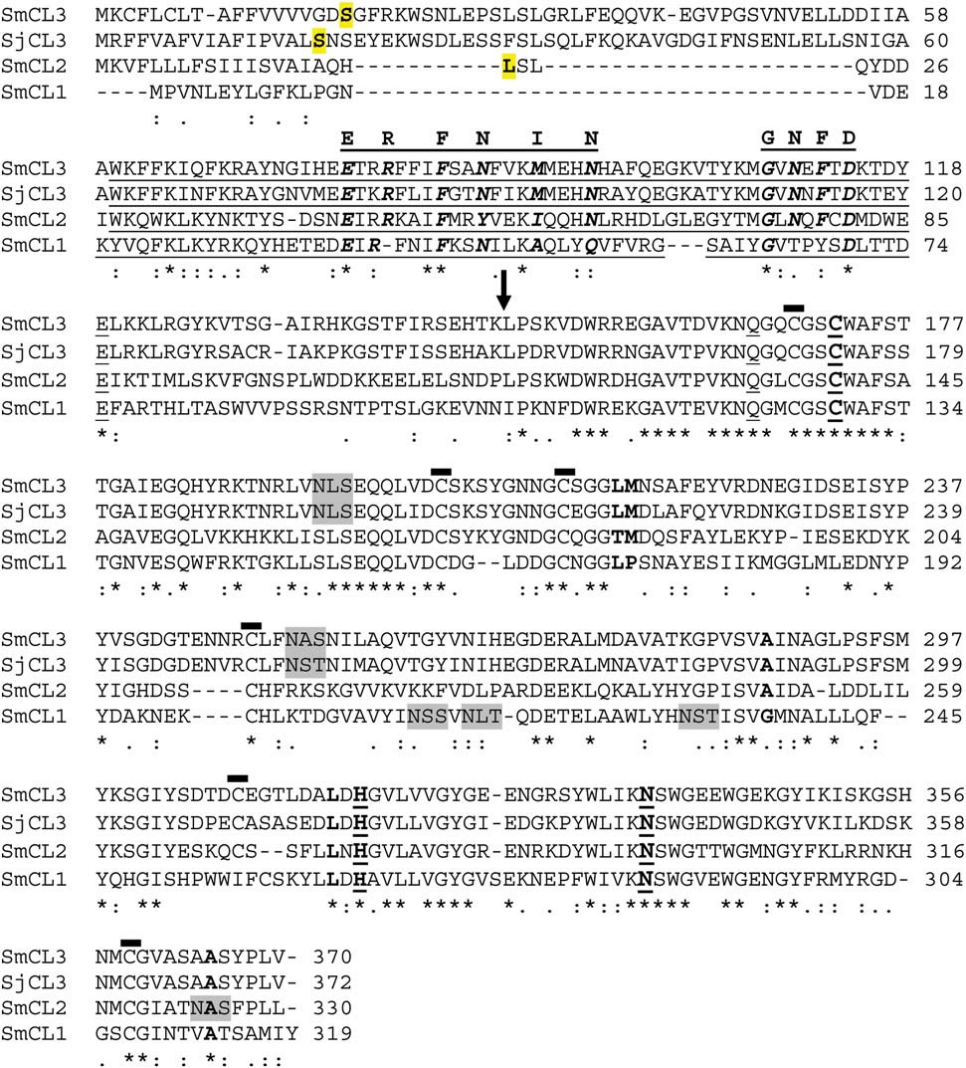
Multiple alignment of SmCL3 (ABV71063) with orthologous SjCL3 from *S. japonicum* (AAW27185) and characterized *S.mansoni* cathepsin L-like genes (SmCL2, CAA83538; SmCL1, Q26534). The catalytic triad residues (C, H and N) are marked in bold and underlined. Glutamine involved in the formation of the oxyanion hole and preceding the catalytic cysteine is underlined. Potential N-linked glycosylation sites are shaded in grey. The predicted starts of the pro-peptide and catalytic domains are highlighted in bold and shaded in yellow, and by an arrow, respectively. Type I-29 protease inhibitor is underlined. ERFNIN and GNFD motifs present in the pro-peptide are overlined with amino acid residues highlighted in bold italic. Six cysteines forming three putative disulfide bonds that are present the catalytic domain are marked by bold overlines. Residues forming the critical S2 subsite specificity pocket are in bold.

PCR strategies based on EST information [18] led to the amplification, sequencing and characterization of a novel cathepsin L gene in *S. mansoni* that we term SmCL3 in accordance with the previously used nomenclature [7],[46]. PCR screening did not identify other gene isoforms. The open reading frame (ORF) consists of 1113 bp (370 amino acids; GenBank accession EU022371) that encodes a pre-proenzyme (Fig. 1). The signal leader sequence was predicted to have a length of 16 amino acid residues. The 130 residue pro-peptide sequence was predicted from a multiple sequence alignment using BLASTP 2.2.18 (http://www.ncbi.nlm.nih.gov/blast/) [38]. Mw/pI values, calculated by the Compute pI/Mw program (http://www.expasy.org) [47], are 41.2/6.5, 39.4/6.5, and 24.1/4.9 kDa for the full length, zymogen and mature proteins, respectively. Cys^172^, His^317^, Asn^337^ form the protease's catalytic triad that is essential for peptidolytic activity. Gln^168^, a residue expected to be involved in the formation of the oxyanion hole, is present. The mature (catalytic) domain has 3 putative disulfide bonds typical of other cathepsin L enzymes [48]. Potential N-linked glycosylation sites are at positions 194 and 252 (Fig. 1).

Compared to typical cathepsins L, the pro-peptide of SmCL3 is unusually long with an N-terminal extension of approximately 30 amino acids, similar to the *S. japonicum* ortholog (SjCL3; GenBank AAW27185; [49]) and two more distant *Clonorchis sinensis* cathepsins L (Genbank ABK91809, ABJ89815; Hu et al, unpublished). Also, an asparagine residue, present in the pro-peptide of previously characterized *S. mansoni* proteases and a site of *trans*-activation by asparaginyl endopeptidases [30],[50] is absent in SmCL3. Like other cathepsins L, the pro-peptide contains a type I-29 protease inhibitor motif [51],[52] (Fig. 1). A variant of the ERFNIN motif, found in other cathepsin L family pro-peptides [53], is present as ERFNMN. A second motif, GNFD, which is involved in intramolecular processing of other Clan CA proteases [54], is also present in the pro-peptide (Fig. 1). The elongated prodomain is not random coil but is predicted to be alpha helix by protein modeling using Maestro.

### SmCL3 is expressed as a fully processed and activated enzyme by *P. pastoris*

**Figure 2:**
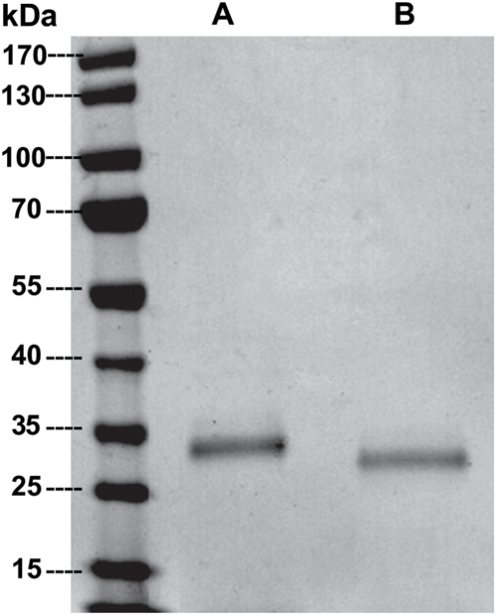
Recombinant SmCL3 expressed in *Pichia pastoris.* (A) SmCL3 after purification by nickel-affinity chromatography was resolved by SDS-PAGE analysis (4–20%) and stained by SimplyBlue SafeStain. Lanes A and B represent recombinant enzyme before and after treatment with Endo-H glycosidase, respectively.

**Figure 3:**
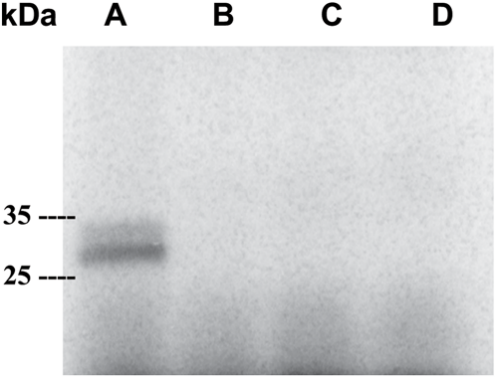
Active site labeling of recombinant SmCL3 by the Clan CA cysteine protease affinity label ^125^I-DCG-04. (A) Purified enzyme incubated with ^125^I-DCG-04. In order to confirm the specificity of the probe to the protease active site, enzyme was (B) incubated prior to labeling with the Clan CA inhibitor E-64, or (C) preheated at 70°C. Sample not incubated with the affinity probe (D). Samples were analyzed by SDS-PAGE (15%) and visualized in phosphor image mode.

SmCL3 was successfully expressed in the yeast *P. pastoris*, fully processed and activated; i.e., without the presence of the pro-peptide. Typical yields of recombinant SmCL3 were 30–50 mg/l of expression media. Peptidolytic activity was evident with or without the C-terminal 6×His-tag using the dipeptidyl substrate Z-Phe-Arg-AMC. As judged by kinetic analyses and assays with the positional scanning synthetic combinatorial library (see SmCL3 positional scanning below), the presence of this 6×His-tag had no effect on catalysis and this expression variant was, therefore, used for subsequent studies. The Clan CA specific inhibitor, E-64, eliminated peptidolytic activity, thus verifying the catalytic mechanism as consistent with cysteine proteases. Using SDS-PAGE, the estimated molecular mass was ∼32–34 kDa which decreased to 28–30 kDa after enzymatic deglycosylation (Fig. 2) consistent with the use of at least one of the two potential glycosylation sites by *Pichia*. Purified SmCL3 was labeled with the cysteine protease affinity probe, ^125^I-DCG-04 (Fig. 3) and cleaved gelatin on zymogram gels (not shown). Though the enzyme was expressed as fully active, some processing heterogeneity was noted by N-terminal protein sequencing of the purified expression product. The most abundant cleavage site (as predicted above) was after the Lys^153^ (HTK^↓^LPS, Fig. 1). A less abundant and slower migrating protein species was also produced by *Pichia* (Fig. 3). ^125^I-DCG-04-labeling confirmed the band as a variant form of SmCL3 (Fig. 3). We attempted to sequence this minor band but without success.

### SmCL3—proteolytic activity and specificity

**Figure 4:**
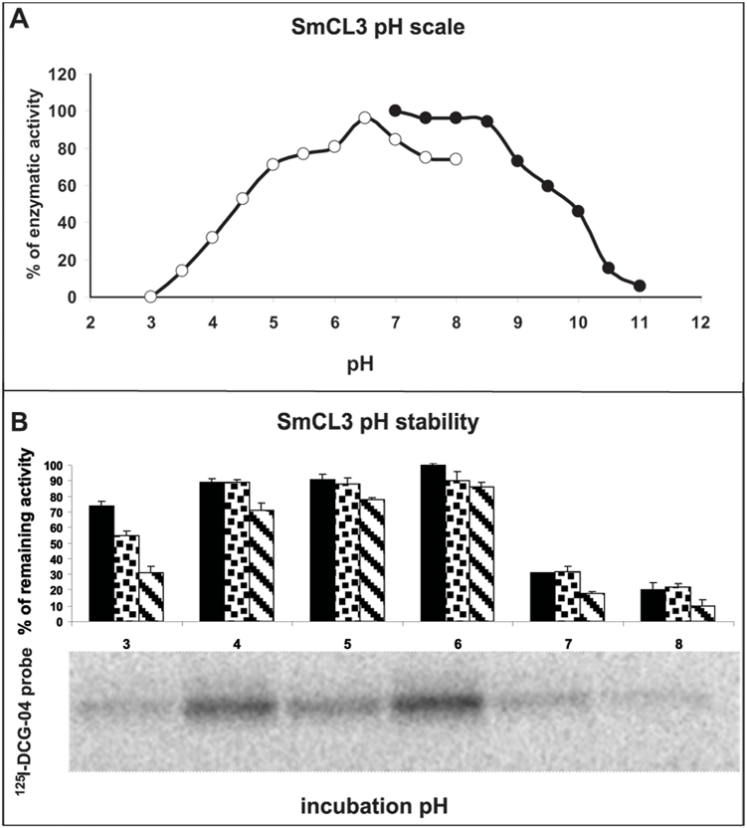
pH optimum and stability of recombinant SmCL3. (A) Using the fluorogenic peptidyl substrate, Z-Phe-Arg-AMC, SmCL3 demonstrates a broad pH optimum. Enzyme was assayed in 50 mM citrate, 100 mM sodium phosphate buffer (○) or 100 mM glycine buffer (•), both containing 2 mM DTT. (B) Peptidolytic activity was measured with Z-Phe-Arg-AMC in 50 mM citrate, 100 mM sodium phosphate, 2 mM DTT, pH 6.0, after preincubation under different pH conditions in 50 mM citrate, 100 mM phosphate for 30 min (black bars), 1 h (dotted bars) and 3 h (striped bars). Samples that had been incubated for 3 h were also labeled with the active site affinity probe ^125^I-DCG-04 (amount of active enzyme visualized corresponds to the amount measured with Z-Phe-Arg-AMC).

**Figure 5:**
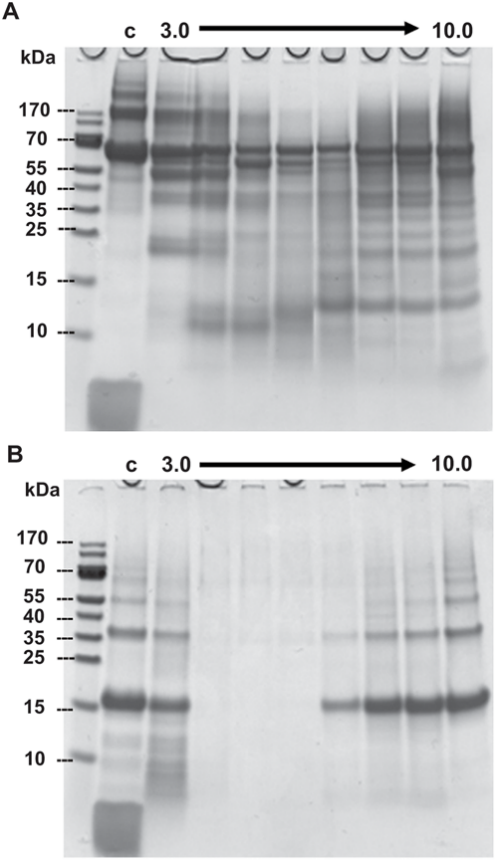
Hydrolysis of bovine serum albumin and hemoglobin by SmCL3. Recombinant SmCL3 (∼100 nM) was incubated with serum albumin (A) or bovine hemoglobin (B) overnight at 37°C in 50 mM citrate, 100 mM sodium phosphate (pH 3.0–7.0) or 100 mM glycine (pH 8.0–10.0.) buffers containing 2 mM DTT. Some cleavage can be observed across the whole pH spectrum. Cleavage was optimal for both protein substrates between pH 4.0–7.0. (C) Samples incubated at pH 5.0, but without the presence of protease.

SmCL3 is catalytically active over a broad pH range. Hydrolysis of Z-Phe-Arg-AMC displayed a bell-shaped pH profile from pH 3.0–11.0 with optimal activity around pH 6.5 (Fig. 4A). Bovine albumin and bovine hemoglobin were degraded: albumin was partially hydrolyzed with a pH optimum around 6.0 (Fig. 5A); hydrolysis of hemoglobin was complete at pH 4.0–6.0 with partial hydrolysis at lower and higher pH values (Fig. 5B). These pH dependencies for activity correlated with the enzymatic stability of SmCL3 between pH 4.0–6.0 as measured with both Z-Phe-Arg-AMC and ^125^I-DCG-04 (Fig. 4B). No loss of activity was recorded after incubation of enzyme for 30 min, 1 and 3 h. However, at other pH values, a time dependent decrease in activity was measured. Therefore, the difference in profiles between the activity and stability experiments is possibly due to the instability of the enzyme at pH values equal to or greater than 7.0.

Kinetic constants obtained for SmCL3 with Z-Phe-Arg-AMC were: *K*_m_ = 20.2 µM and *k*cat/*K*m = 410 mM^−1^ s^−1^. Inhibition constants (*k*_obs_ at 1 nM of inhibitor) measured for SmCL3 with E-64 and K11777 were 26.5 and 140 nM^−1^ s^−1^, respectively.

**Figure 6:**
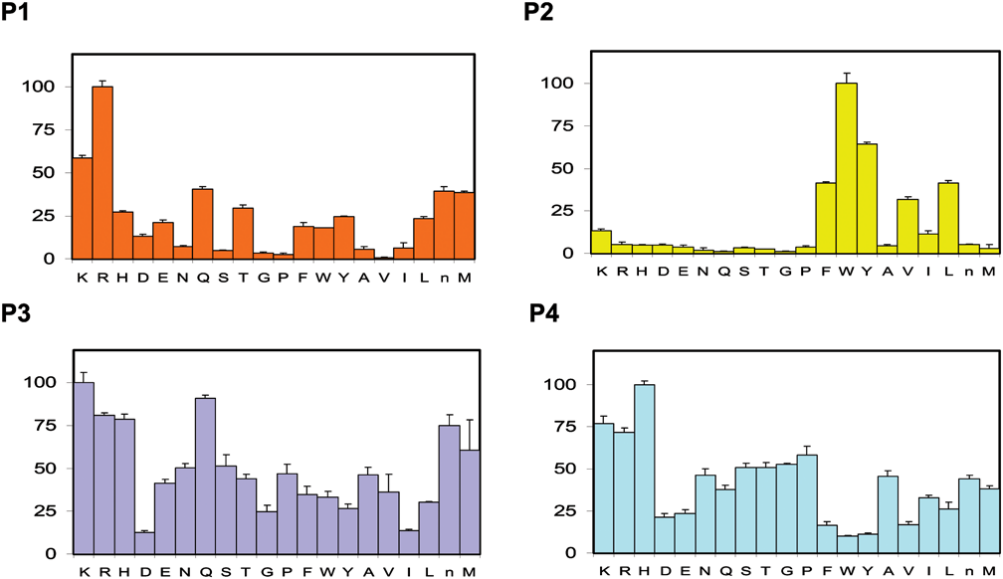
P1–P4 specificity profile of SmCL3 using positional scanning-synthetic combinatorial libraries. The P2 substrate position shows the strongest preference for specific amino acid types with large hydrophobic residues being most favored. Y-axis represents % of preference for the particular amino acid when 100% represents most preferred residue.

Consistent with other Clan CA proteases [35], SmCL3 prefers the basic amino acids lysine and arginine at the P1 subsite position (Fig. 6). At P2, the enzyme prefers hydrophobic amino acids, especially bulky aromatic residues. Upon a search of the literature involving PS-SCL assays, the P2 preferences of SmCL3 was found to closely resemble those of human cathepsin V [35], a cathepsin L-like protease. In particular, there is an overriding preference for tryptophan and equal preference for phenylalanine and leucine in the P2 sites of both enzymes. Screening at P3 and P4 revealed greater promiscuity. Notably, SmCL3 is able to accept aspartic acid at P2 and P3 positions, which is similar to human cathepsin F [35].

### The 3D structural model of SmCL3 identifies a large and deep S2 subsite pocket and secondary structure predictions indicate a helical type prodomain

**Figure 7:**
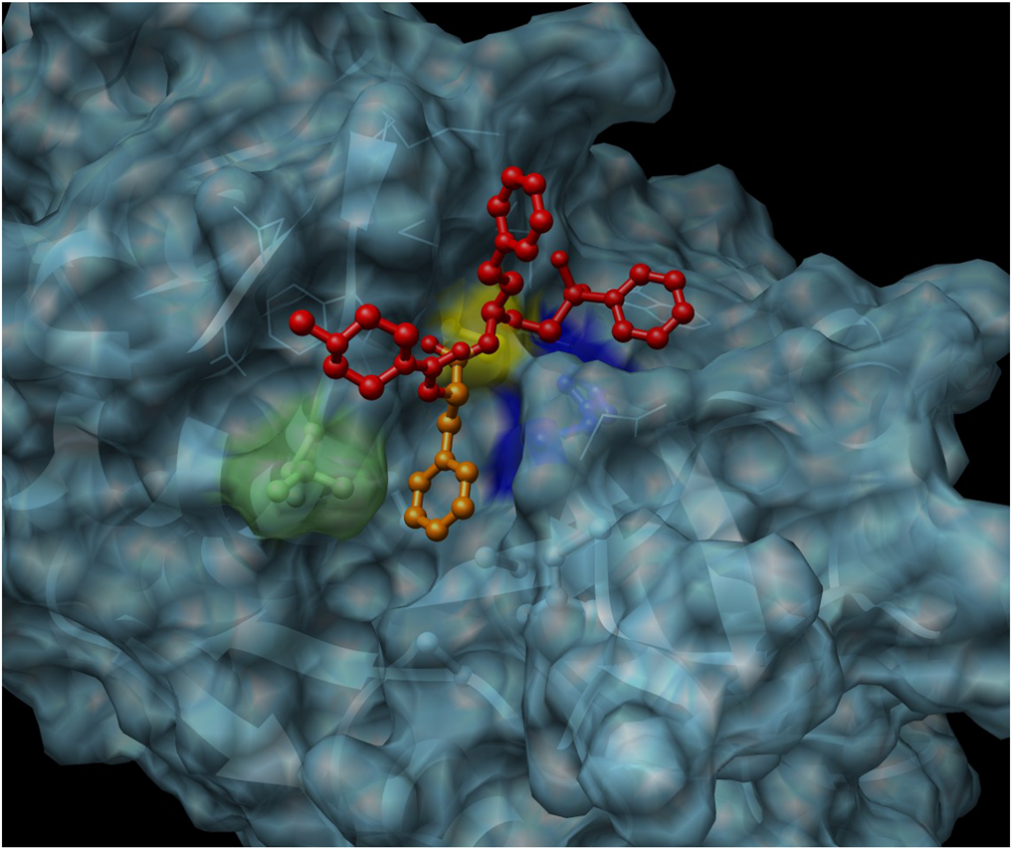
Structural model of SmCL3 in complex with the peptidyl vinyl sulfone inhibitor, K11777. The inhibitor is shown in red and orange; the moiety that interacts with the S2 pocket is in orange. The catalytic Cys^172^and His^317^ residues are colored yellow and blue, respectively. The predicted residues in the deep S2 binding pocket are identical to those in human cathepsin V (used as a template for the model) except for a leucine residue (Leu^216^, colored light green).

For the three-dimensional model of SmCL3, the X-ray crystallographic structure of human cathepsin V complexed with a peptidyl vinyl sulfone inhibitor, K11777, was used as template. We used this template because of its high percentage identity (59%) to SmCL3 and because the structure was solved with an inhibitor in the active site thereby likely making the modeling of the active site more accurate. From the structure-based alignment, the predicted interaction of the modeled structure with K11777 is depicted in Fig. 7. The predicted residues in the S2 binding pocket of SmCL3 are identical to those in cathepsin V with the exception of a Leu residue (Leu^216^) which is phenylalanine (Phe^69^) in cathepsin V. This substitution enlarges what is already a deep and wide pocket, and consistent with the results from the PS-SCL, appears well adapted to accept the side chains of bulky aromatic residues, such as tryptophan, tyrosine and phenylalanine.

The prodomain region was lacking in the template and so is not included in the model. However, secondary structure prediction indicates that five helices are likely to form in the SmCL3 prodomain (not shown).

### SmCL3 is mainly expressed as a zymogen in the gut of the parasite stages infecting the definitive host

**Figure 8:**
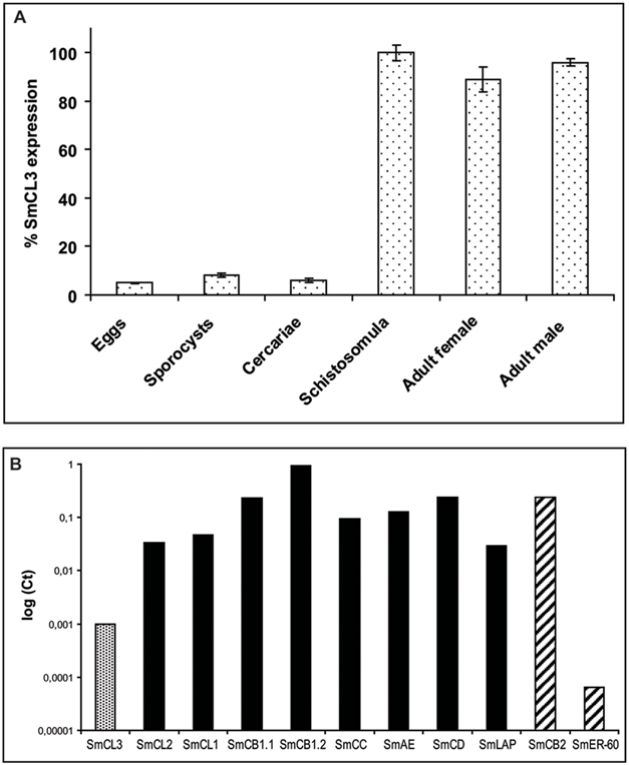
Developmental regulation of SmCL3 transcription and transcript abundance relative to other *S. mansoni* proteases. (A) The highest CT value (which was 0.94) was set as 100%. (B) In the adult worms (mixed sexes) CT levels of mRNA encoding SmCL3 (dotted bar) were compared with gut-associated proteases (black bars) and two other proteases, the tegumental/parenchymal SmCB2 and endoplasmatic reticulum-associated SmER-60 (striped bars). Standard deviations were never greater than 0.3 for initial CT values. For enzyme nomenclature [7],[46] see Introduction.

Quantitative PCR demonstrated that SmCL3 is predominantly expressed in those developmental stages infecting the mammalian host (Fig. 8A), a result that is in accord with the protein expression profile as shown by immunoblots with specific polyclonal anti-SmCL3 IgG (see below). Most mRNA for SmCL3 was detected in transformed schistosomula *in vitro*, and adult male and female worms. Expression profiling by qPCR indicated that SmCL3 mRNA is 50 to 1000 fold less abundant relative to previously described gut-associated proteases in *S. mansoni* adults [7],[8] (Fig. 8B). SmCL3 mRNA is also less abundant than that of the tegumental/parenchymal SmCB2 [27] (more than 100-fold), but is found in greater quantities than the endoplasmatic reticulum protease, SmER-60 [55] (more than 10-fold).

**Figure 9:**
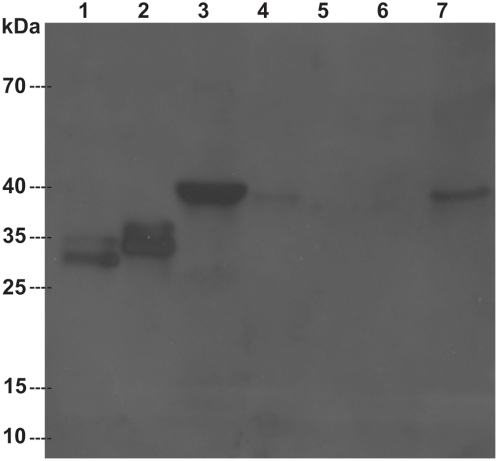
Detection of SmCL3 by Western blot in soluble *S. mansoni* extracts using mouse polyclonal IgG antibodies. Recombinant enzyme or soluble *S. mansoni* protein extracts (each 20 µg) were resolved by SDS-PAGE (15%) and electroblotted onto PVDF membrane. IgG purified antibodies reacted with (1) deglycosylated recombinant protein, (2) glycosylated protein, soluble extracts of (3) adults, (4) eggs and (7) 1 day old *in vitro* transformed schistosomula. Extracts of (5) miracidia and (6) cercariae did not react.

By immunobloting with specific polyclonal anti-SmCL3 IgG (Fig. 9), native SmCL3 was detected in extracts of both adults and newly-transformed schistosomula 1 h after *in vitro* transformation. Weaker reactivity was detected in extracts of eggs and no reaction was found in extracts of miracidia and cercariae. Control mouse IgG antibodies were non-reactive throughout (not shown).

**Figure 10:**
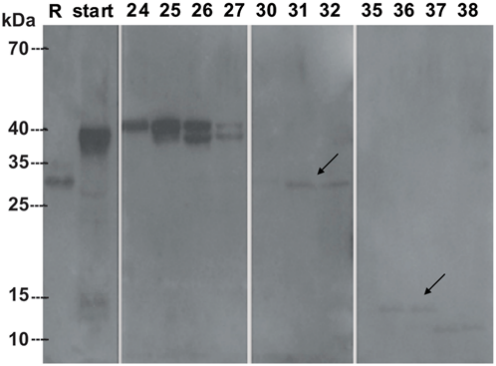
Western blot analysis of SmCL3 in adult *S. mansoni* soluble protein extract after size exclusion chromatography. Several protein species were detected after size exclusion chromatography. Two species corresponding to the molecular mass of the zymogen are present in fractions 24–27. A protein species of 28 kDa corresponding to the mass of the mature enzyme is recognized in fractions 30–32. Two species of 15 and 13 kDa was detected in fractions 35–38. R - recombinant glycosylated SmCL3 electroblotted as a control; start - soluble protein extract (∼60 µg) before size exclusion chromatography.

Unlike the immuno-reactivity observed at approximately 30 kDa for the recombinant enzyme (Fig. 9, lanes 1 and 2), the major reactive band in schistosome extracts migrated with a mass of approximately 40 kDa (Fig. 9, lanes 3, 4 and 7), a mass that corresponds to that of the SmCL3 zymogen. Attempts to process *in trans* pro-SmCL3 within extracts using other recombinant proteases such as SmCB1 [30] and a asparaginyl endopeptidases from tick [36] or *S. mansoni* [30] failed, as did incubation of worms extracts overnight at 37°C in an effort to endogenously process the zymogen (not shown). The data suggest, therefore, that SmCL3 is present in its major form as a zymogen rather than as mature catalytically active enzyme. As judged by immunoblotting, size exclusion chromatography of *S. mansoni* adult soluble protein extracts separated the putative SmCL3 zymogen (Fig. 10, fractions 24–27) from the immunoreactive protein species of 30 kDa – the possible mature enzyme (fractions 30–32), and of 13 and 11 kDa – possible SmCL3 fragments (fractions 35–38).

SmCL3 was not detected by specific polyclonal IgG in excretory/secretory (E/S) products of adult worms maintained in culture medium. Nevertheless, SmCL3 was detected by antibody in the regurgitant when adult worms were induced to regurgitate in water (data not shown).

**Figure 11:**
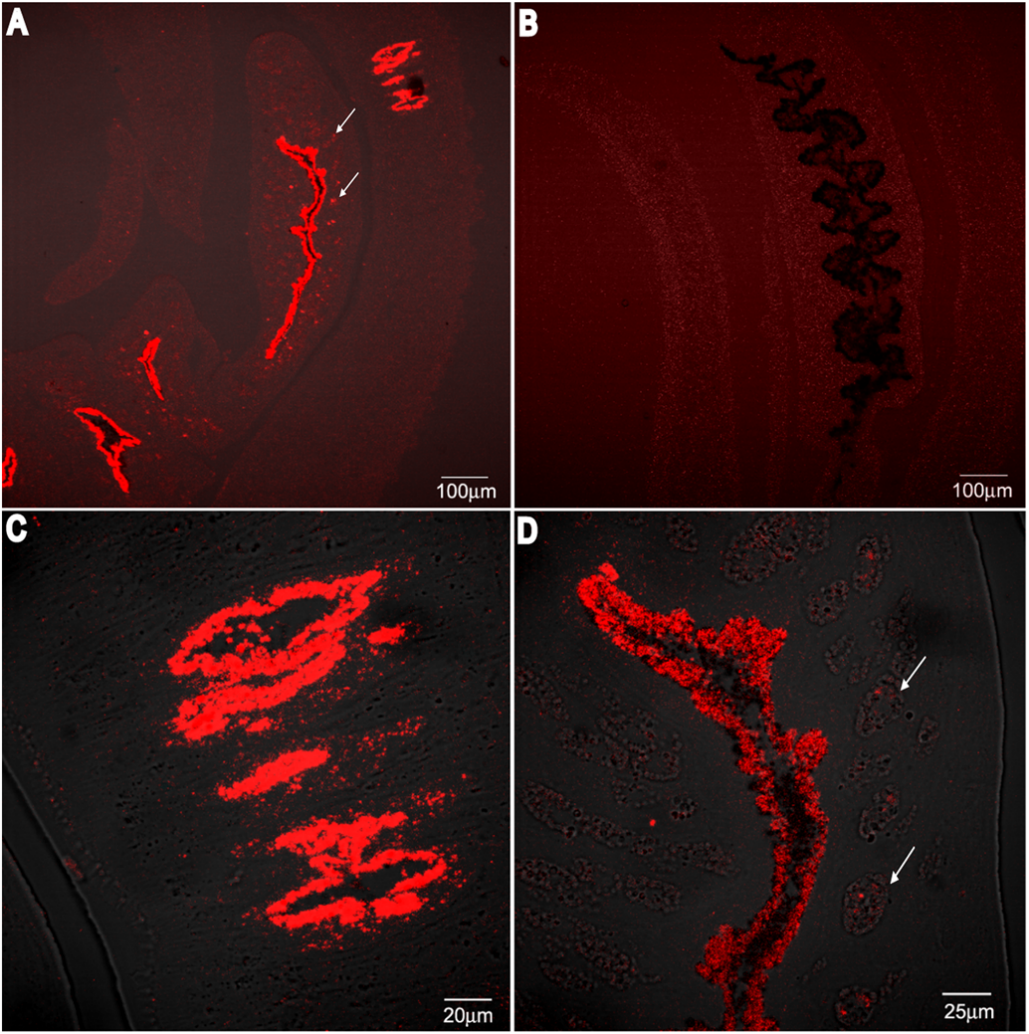
Localization of SmCL3 in adult worms by immunofluorescence confocal microscopy. Mono-specific mouse IgG and subsequent amplification with Alexa Fluor 594 anti-mouse IgG were used to localize SmCL3. A strong reaction is detected in the gastrodermis of *S. mansoni* males and females (A). In males (C), the reaction is apparently exclusive to the gastrodermis whereas in females (D) a reaction is also noted in the vitellaria (white arrows). Control mouse IgG antibodies did not react even after over-exposure of the image (B).

SmCL3 was localized to the gastrodermis of both adult sexes with some reaction in the female vitellaria using confocal microscopy with mouse anti-SmCL3 IgG and Alexa Fluor 594 secondary antibodies (Fig. 11A, 11C, and 11D). No reaction was observed in the tegument and parenchyma. No staining was observed with control mouse polyclonal IgG (Fig. 11B).

### Sequence similarity clustering of cathepsins L recapitulates taxonomic kingdom groupings with SmCL3 among the Opisthokonta

**Figure 12:**
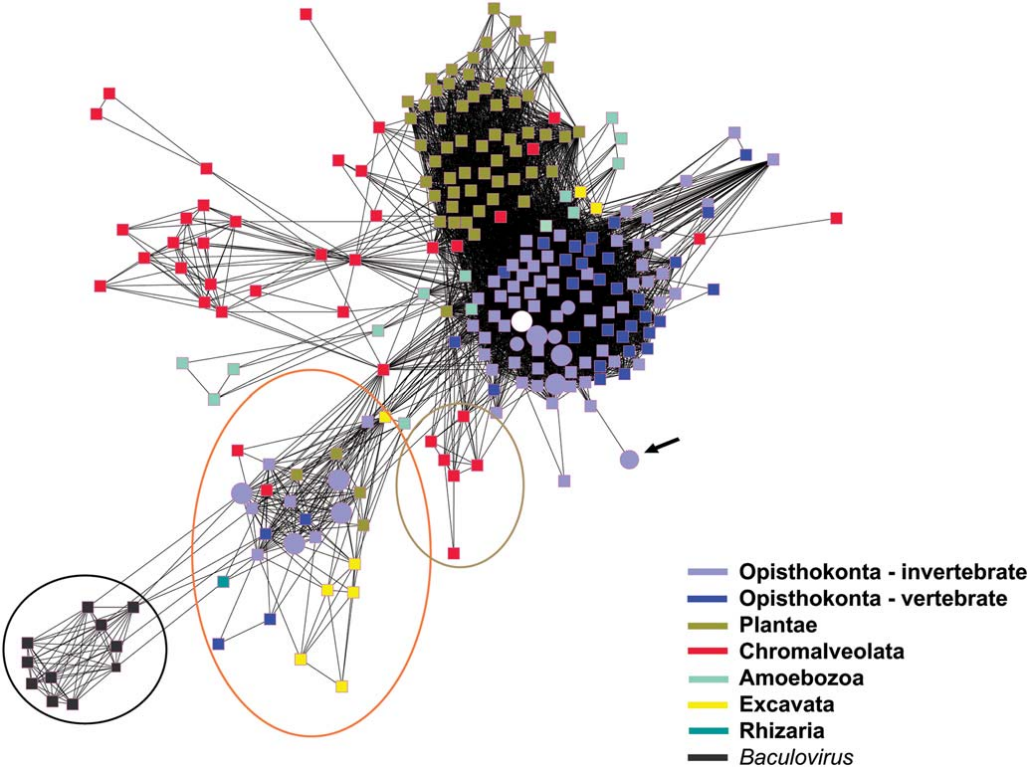
Sequence relationships among the cathepsin L enzymes. Clustering of cathepsin L sequences accords with the organization of the kingdoms of life into six super kingdoms [45]: Opisthokonta (light purple and dark blue nodes), Plantae (green), Chromalveolata (red), Amoebozoa (aquamarine), Rhizaria (single turquoise node) and Excavata (yellow). Circular nodes represent groups of cathepsin L sequences from platyhelminths and those that are enlarged identify cathepsin L sequences specific to Trematoda. Square nodes depict cathepsin L sequences from other groups. The white circle represents the sequence from SmCL3 and its *S. japonicum* ortholog, SjCL3, which are found among the opisthokont, invertebrate metazoan cathepsin L genes. The large circular node more distant from the main opisthokont cluster corresponds to an inactive cathepsin L ortholog from *S. japonicum* (black arrow). The network view identifies a diverse group of cathepsin L sequences making up a cluster enriched in cathepsin L subtypes cathepsins F and W (encircled in orange) that includes sequences from *S. mansoni*, *S. japonicum*, *O. viverrini*, *C. sinensis*, *P. westermani* and *M. yokogawai*. Encircled in green are sequences exclusive to the cathepsin H subtype of cysteine cathepsins L. Finally, a cluster composed solely of baculovirus cathepsin L genes (within the black circle) is least connected to all other sequences in the network.

A network view of primary protein sequence similarity relationships among cathepsin L type enzymes was generated using the software Cytoscape [19]. Each sequence is represented as a square node, except for cathepsin L sequences from platyhelminths which are indicated by circular nodes and those representing Trematoda are enlarged circular nodes (Fig. 12). Of immediate interest is that the clustering of cathepsin L sequences agrees closely with the taxonomic organization of the kingdoms of life into six supergroups [45]: Opisthokonta, Plantae, Chromalveolata, Amoebozoa, Rhizaria and Excavata (Fig. 12). SmCL3 (white circular node) is found within a large cluster of closely related invertebrate (light blue squares) and vertebrate metazoan (dark blue squares) cathepsins L that together make up the super kingdom Opisthokonta. This large cluster also includes the *S. japonicum* ortholog, SjCL3, two *C. sinensis* cathepsin L genes, the SmCL2 gene and related cathepsins L from *Fasciola gigantica* and *F. hepatica*. The cluster is distinct from a cluster of cathepsins L that is restricted to the Plantae super kingdom, an organizational level of primary plastid endosymbionts comprising plants, green and red algae. More disparate clusters of cathepsin L sequences are found in the super kingdom Chromalveolata (secondary symbionts; contains apicomplexan parasites such as *Toxoplasma* and *Cryptosporidium*), the Amoebozoa (includes the parasite *Entamoeba histolytica*). Another compact cluster displayed in Fig. 12 is entirely composed of baculovirus cathepsin L-like genes (encircled black) and is least connected to the other clusters.

The Cytoscape view also resolves a cluster of sequences that is enriched in cathepsins F and W, which are subtypes of cathepsin L (encircled in orange). This cluster includes sequences of greater phylogenetic diversity including SmCLl (aka SmCF) [7], cathepsins F from *Opisthorchis viverrini*, *Clonorhis sinensis*, *Paragonimus westermani* and *Metagonimus yokogawai*, and Excavata parasitic kintetoplastid cathepsins. Finally, a small cathepsin H cluster, another subtype of cathepsin L (encircled in green) is resolved that from the clusters containing cathepsins L and F/W. For sequence details see supporting Cytoscape data (Fig. S1; Note: after downloading Cytoscape).

## Discussion

Growth, maturation and fecundity of the schistosome parasite in the mammalian host rely on nutrients ingested from the host bloodstream. A number of proteases are expressed in the gut of *S. mansoni* and are involved in the degradation of hemoglobin and serum proteins [7],[8]. This multienzyme network includes two cathepsins L, SmCL1 (aka SmCF) and SmCL2 [7],[46],[56]. Although sequences for other cathepsins L exist in the EST datasets [18] and in first pass assembly of the genome (Mashiyama, Caffrey, Sajid, unpublished), nothing is known about their contribution to schistosome metabolism. Here, we identified, heterologously expressed and characterized a novel gut-associated cathepsin L that we term SmCL3. A sequence for an ortholog in *S. japonicum* (SjCL3) also exists (GenBank AAW27185; [49]).

SmCL3 possesses sequence characteristics consistent with those of other cathepsins L. These include six Cys residues forming three disulphide bonds [48], an active site catalytic triad of Cys, His and Asn [57], the residue Gln^168^ involved in the formation of the oxyanion hole, a pro-peptide that contains an I29 inhibitor family sub-domain and a variation of the ERFNIN motif (ERFNMN) that is typical for cathepsins L [53]. This motif, together with the motif GNFD [54], is probably involved in intra-cellular trafficking and processing.

Other sequence features of SmCL3 are more unusual, especially when compared to other helminth cysteine proteases associated with the gut. First, an Asn residue, found between the pro-peptide and mature domain of other gut cathepsins in *Schistosoma* [30],[50] and fasciolids [50],[58], and demonstrated to be a processing site for pro-cathepsin activation by an asparaginyl endopeptidase (AE) [30], is absent. Unlike recombinant *S. mansoni* pro-cathepsin B1 expressed in *Pichia* that requires *trans*-processing by an endogenous AE for full activity [30], SmCL3 is already fully processed in *Pichia* induction medium at the predicted cleavage site, as judged by Edman N-terminal sequencing and proteolytic activity. This suggests that recombinant pro-SmCL3 is capable of auto-catalytic activation and maturation. Secondly, the SmCL3 zymogen has an unusually long pro-peptide comprising 130 residues. Approximately the first 30 amino acids of the pro-peptide share some homology with the SmCL3 ortholog in *S. japonicum* and two *C. sinensis* cathepsins L. For the SmCL3 prodomain, five helical structures were predicted which imply some regulatory or supplementary structural role. Prodomains that are extended N-terminally, though different in sequence, are also found in the gut-associated cathepsins L of the animal parasitic nematode *Gnathostoma spinigerum* [59] and the plant parasitic nematode *Meloidogyne incognita* [60]. These extensions may confer additional functionality to the zymogen, perhaps in protein trafficking or as binding sites for other proteins. It is also possible that this extension may be associated with the fact that the major form of the enzyme in the parasite apparently exists as a zymogen and/or the enzyme seems not to be secreted into the gut lumen (see discussion below).

SmCL3 cleaves albumin and hemoglobin most efficiently at pH values between 4.0 and 6.0. The pH dependency of hydrolysis of the Z-Phe-Arg-AMC synthetic substrate results in a bell-shaped curve from pH 3.5 to 11.0 with an optimum at 6.5. At least 40% of total activity can be detected between pH 4.0 and 10.0. A similar bell-shaped pH profile was measured for SmCL1 [46], which, unlike SmCL2, was able to cleave peptidyl substrate at basic pH. The acidic pH optima measured against both protein and peptidyl substrates correlates with the pH of the gut lumen (∼pH 6.5) [30],[61] and with the lower pH (∼4.0) micro-environments thought to form upon fusion of gut lamellae and where it is hypothesized that the bulk of gastrodermal proteolysis by cysteine and aspartic proteases takes place [8].

Recombinant SmCL3 possesses peptidolytic characteristics consistent with its classification as a Clan CA Family C1 protease: it is effectively inhibited by the Clan CA-specific inhibitors E-64 and K11777 [32],[33], and labeled by the affinity probe DCG-04 [29]. Positional scanning using diverse synthetic substrate libraries revealed a typical Clan CA preference profile: no single amino acid preference in S4 and S3 but a strong preference for lysine and arginine in the S1 subsite [35]. However, in S2 (the subsite driving specificity in Clan CA proteases), hydrophobic amino acids (Trp>Tyr>Phe/Val>Leu) are preferred. These preferences at P2 are similar to those of human cathepsin V [35] but differ, for example, from *F. hepatica* cathepsins L1 and L2 that exhibit a singular preference for Leu and Pro in the case of FhCL2 [62],[63]. In support of the S2 preferences demonstrated biochemically with the PS-SCL, the 3D structural model of SmCL3, using K11777 as the bound ligand, visualizes a large and deep S2 pocket. The amino residues forming the S2 pocket are identical to those of cathepsin V with the exception of one substitution of a Leu (residue 216) instead of Phe.

SmCL3 is developmentally regulated at both the mRNA and protein levels being mainly expressed in those stages (schistosomula and adult) infecting the definitive mammalian host and thus suggesting a function(s) particular to these developmental stages. That one of these functions is associated with the digestion of host blood proteins is supported by confocal microscopy using polyclonal IgG that localizes SmCL3 to gastrodermis of adult worms. The hypothesis is consistent with the ability of the enzyme to degrade biologically relevant protein substrates, i.e., hemoglobin and bovine serum albumin, as discussed above. Given that the transcription of SmCL3 is 50 to1000 fold less than other gut-associated proteases, the actual proteolytic contribution by SmCL3 to total proteolysis in the gut remains to be determined.

Because of its localization, it is conceivable that SmCL3 operates with the other gut proteases to complete the degradation of host proteins as nutrients [7],[8]. However, unlike proteases such as SmCB1, SmCL1 and SmCL2 [30],[44],[56], SmCL3 was not detected in worm E/S products when maintained in isotonic culture medium. However, when adult worms were induced to regurgitate in water, i.e., exposed to hypotonicity, potentially causing damage to gut cells, SmCL3 could then be detected by specific antibody in the regurgitant (data not shown). This suggests that SmCL3 is normally retained within the gastrodermal epithelium and is not secreted. Of note is that the *G. spinigerum* cathepsin L is likewise not detected in E/S products even though it is found in the gastrodermis [59]. Apart from the gut, some immuno-reaction for SmCL3 was also noted in the vitellaria of female *S. mansoni*, a finding consistent with the presence of a small amount of SmCL3 in eggs by immunoblotting. Therefore, SmCL3 may also function in egg and/or miracidial metabolism.

By immunoblotting, SmCL3 was detected in parasite extracts at a molecular mass of approximately 40 kDa, i.e., consistent with that of the zymogen. A similar situation was noted recently for the *G. spinigerum* cathepsin L – the major species of that enzyme also migrated at approximately 40 kDa in worm extracts [59]. Attempts to *trans*-process the SmCL3 zymogen in worm extracts using other recombinant proteases failed, as did incubating worm extracts overnight at 37°C. After size-exclusion chromatography of adult worm extracts, in addition to the resolution of a major 40 kDa protein, minor immuno-reactive protein species were detected at approximately 28, 15 and 13 kDa. These may represent the mature deglycosylated enzyme, and two degradation products, respectively. It seems, therefore, that the major form of SmCL3 in *S. mansoni* is a zymogen. The retention of the pro-peptide with the mature domain opens the possibility of a distinct function(s) for the zymogen, including the possibility of a limited or discrete processing activity against protein and peptide substrates. Precedents for protease zymogens that exhibit peptidolytic activity exist [30],[64]. Often, the strength of the association between the pro-peptide and mature domain is pH dependent [65], allowing access to and cleavage of small peptide substrates.

To investigate evolutionary relationships of the full SmCL3 protein sequence and its cathepsin L neighbors, we examined the top 1,000 hits in a BLAST search of the SmCL3 protein sequence. We found only 16 other trematode sequences for which the N-terminus extended far enough to overlap at least 75% of the prodomain of SmCL3. Most of these sequences were highly similar to each other and after filtering to 90% identity, there were only 5 sequences including the SmCL3 sequence. As expected, a multiple sequence alignment of these sequences showed a highly conserved catalytic domain, and a more variable prodomain region (data not shown). In order to visualize more distant relationships between SmCL3 and cathepsin L-like sequences, we constructed a SSN using the program Cytoscape [19]. We have recently established that SSNs show good agreement with information provided by phylogenetic trees and allow a clear view of all of the represented proteins in a dataset together with easy associations to functional and other types of information [44].

Based on full-length sequences, and as visualized with the software Cytoscape (Fig. 12), the sequence clustering of cathepsin L proteins recapitulates the recent proposed partition of Eukarya into six “super kingdoms” based on multivariate phylogenetic analyses [45]. SmCL3 is found within the Opisthokonta super kingdom (that includes animals and fungi). The Opisthokonta cathepsins L also includes the SjCL3 ortholog from *S. japonicum*, SmCL2 [66], and two *C. sinensis* cathepsins L (Hu et al, unpublished), as well as a collection of sequences from *F. hepatica* and *F. gigantica* [58]. The network clearly resolves a cluster that is enriched in cathepsins F/W (encircled in orange) and another enriched in cathepsin H (encircled in green). This confirms the previous distinction of the subgroup cathepsins F/W from the main body of cathepsins L, which arose as a result of gene fusion between an ancestral cathepsin L and a cystatin (cysteine protease inhibitor) [67], and from another cathepsin L subgroup, cathepsins H, which contain a specific mini-chain formation to function as aminopeptidases [68]. The inclusion within the cathepsin F/W cluster of SmCL1 (SmCF), other trematodal cathepsins F and the kinetoplastid cathepsins L supports and extends previous phylogenetic data [48], [69]–[71]. Overall, given the close agreement of the distance relationships observed here between cathepsins L and the taxonomic separation of the tree of life proposed previously [45], we would suggest that cathepsins L are useful ‘marker genes’ for inclusion in future phylogenetic analyses. More in-depth studies of this and other issues in a global analysis of the members of this super-family may be enlightening future work.

To conclude, SmL3 is a gut-associated protease with some unusual sequence and biophysical features. The enzyme may function as part of the network of proteases [8] that facilitates the digestion of host proteins by the schistosome parasite. As inhibitors of Clan CA proteases are therapeutic in animal models of schistosomiasis [15],[16], it is possible that the inhibition of SmCL3, either alone or in concert with other cysteine proteases, may prove clinically beneficial.

## Supporting Information

Figure S1 Cystoscape analysis file containing sequences. You have to download Cytoscape v2.4.1 program at http://www.cytoscape.org/index.php
(0.13 MB ZIP)

Table S1 List of primers used for qPCR analysis
(0.05 MB DOC)
