# Supplementary material for: SmCL3, a Gastrodermal Cysteine Protease of the Human Blood Fluke Schistosoma mansoni
Source: PLoS Negl Trop Dis. 2009 Jun 2;3(6):e449. doi: 10.1371/journal.pntd.0000449 (PMC2685030; doi:10.1371/journal.pntd.0000449)
Supplement: Table S1 — List of primers used for qPCR analysis (0.05 MB DOC) [file pntd.0000449.s002.doc]

| **Primer set** | **Forward Primers** | **Reverse primers** |
| --- | --- | --- |
| **Aqrt-SmCL3** | CGGTCGGCTCATTTAGTCAT | TCCAAGCTGCAATAATGTCG |
| **Bqrt-SmCL3** | TGCTGGTCTTCCCAGTTTTT | CCCACTCTTCACCCCAACTA |
| **Aqrt-SmCL2** | gttgctatcgcccaacactt | tcctcccagtccatatcaca |
| **Bqrt-SmCL2** | tgtgatatggactgggagga | cccaacatgatccacacaaa |
| **Aqrt-SmCL1** | aaggcgccgtaactgaagta | ctaaaccgtcgcagtcaaca |
| **Bqrt-SmCL1** | ttcgcacgcactcatctaac | gttttgcggaaccactgact |
| **Aqrt-SmCB1.1** | ACTTGGTGGGCACGCTATAC | TAATTCGACCGGCTGTTACC |
| **Bqrt-SmCB1.1** | ACTTGGTGGGCACGCTATAC | GGCTGTTACCTCGGATTCAA |
| **Aqrt-SmCB1.2** | GAGGAAGAGACGTCCCACAG | TTCGATCACTCATTGCTTCG |
| **Bqrt-SmCB1.2** | CCCATCTCGACGCTCATATT | CTGTGGGACGTCTCTTCCTC |
| **Aqrt-SmCC** | acaccgattcgtaaccaagg | atccttcggagtaggggcta |
| **Bqrt-SmCC** | tgcggtttacatgtgctgat | ccaatgccctctatttccaa |
| **Aqrt-SmAE** | gctggtgggaaagttttgaa | tggaacctgattcacatgct |
| **Bqrt-SmAE** | accgggtctaattgctttcc | agcagctgtagtcgcgtaaa |
| **Bqrt-SmCD** | ATTGGTACGCCACCTCAGAC | AGCCCAACTGAAGGGAATCT |
| **Eqrt-SmCD** | GGTACGCCACCTCAGACATT | ACCATTCGGGATGTAAGTGG |
| **Aqrt-SmLAP** | ttcccggtctcttgtttgac | tgggaatggaatcacactga |
| **Bqrt-SmLAP** | gttcccggtctcttgtttga | tgggaatggaatcacactga |
| **Aqrt-SmCB2** | gaggccaatacgacttggaa | ctaccctcgcatcaaagctc |
| **Bqrt-SmCB2** | catcgtgtggttcttgttgg | aatgtgggaagccaccatta |
| **Aqrt-SmER60** | ctgttttctatccccctgcat | ggtacccgaccaaaagaaca |
| **Bqrt-SmER60** | cccgtacgcttcctgtgata | ttagccaatgtgacgggata |
